# An intron endonuclease facilitates interference competition between co-infecting viruses

**DOI:** 10.1126/science.adl1356

**Published:** 2024-07-04

**Authors:** Erica A. Birkholz, Chase J. Morgan, Thomas G. Laughlin, Rebecca K. Lau, Amy Prichard, Sahana Rangarajan, Gabrielle N. Meza, Jina Lee, Emily Armbruster, Sergey Suslov, Kit Pogliano, Justin R. Meyer, Elizabeth Villa, Kevin D. Corbett, Joe Pogliano

**Affiliations:** 1 Department of Molecular Biology, University of California, San Diego, La Jolla, CA; 2 Department of Ecology, Behavior and Evolution, University of California, San Diego, La Jolla, CA; 3 Department of Cellular and Molecular Medicine, University of California, San Diego, La Jolla, CA; 4 Howard Hughes Medical Institute, University of California, San Diego, La Jolla, CA

## Abstract

Introns containing homing endonucleases are widespread in nature and have long been assumed to be selfish elements that provide no benefit to the host organism. These genetic elements are common in viruses, but whether they confer a selective advantage is unclear. Here we studied an intron-encoded homing endonuclease in bacteriophage ΦPA3 and found that it contributes to viral competition by interfering with the replication of a co-infecting phage, ΦKZ. We show that gp210 targets a specific sequence in ΦKZ, which prevents the assembly of progeny viruses. This work demonstrates how a homing endonuclease can be deployed to engage in interference competition among viruses and provide a relative fitness advantage. Given the ubiquity of homing endonucleases, this selective advantage likely has widespread evolutionary implications in diverse plasmid and viral competition as well as virus-host interactions.

## Summary

A homing endonuclease provides a relative fitness advantage during viral competition.

Mobile introns are widespread in all kingdoms of life (1–4). They are composed of a self-splicing intron, often group I or group II, and a homing endonuclease (5, 6). Homing endonucleases recognize and cleave specific DNA target sequences that are homologous to the region in which its gene was inserted. Cleavage triggers recombination with the homologous chromosome, resulting in unilateral gene conversion and the loss of the target site (6, 7). The mobility of the intron allows it to insert into homologous sites in the genome or a related genome. Phage commonly contain mobile introns (6, 8) capable of invading the unoccupied locus in a related genome, the intron(−) allele. While mobile introns are ubiquitous and have been shown to have a positive influence on the rate of inheritance of neighboring viral genes in processes called coconversion and marker exclusion (9–14), it remains unclear whether these genetic elements are wholly selfish or whether they also provide a competitive advantage to their host (13, 15, 16).

**Figure 1.**
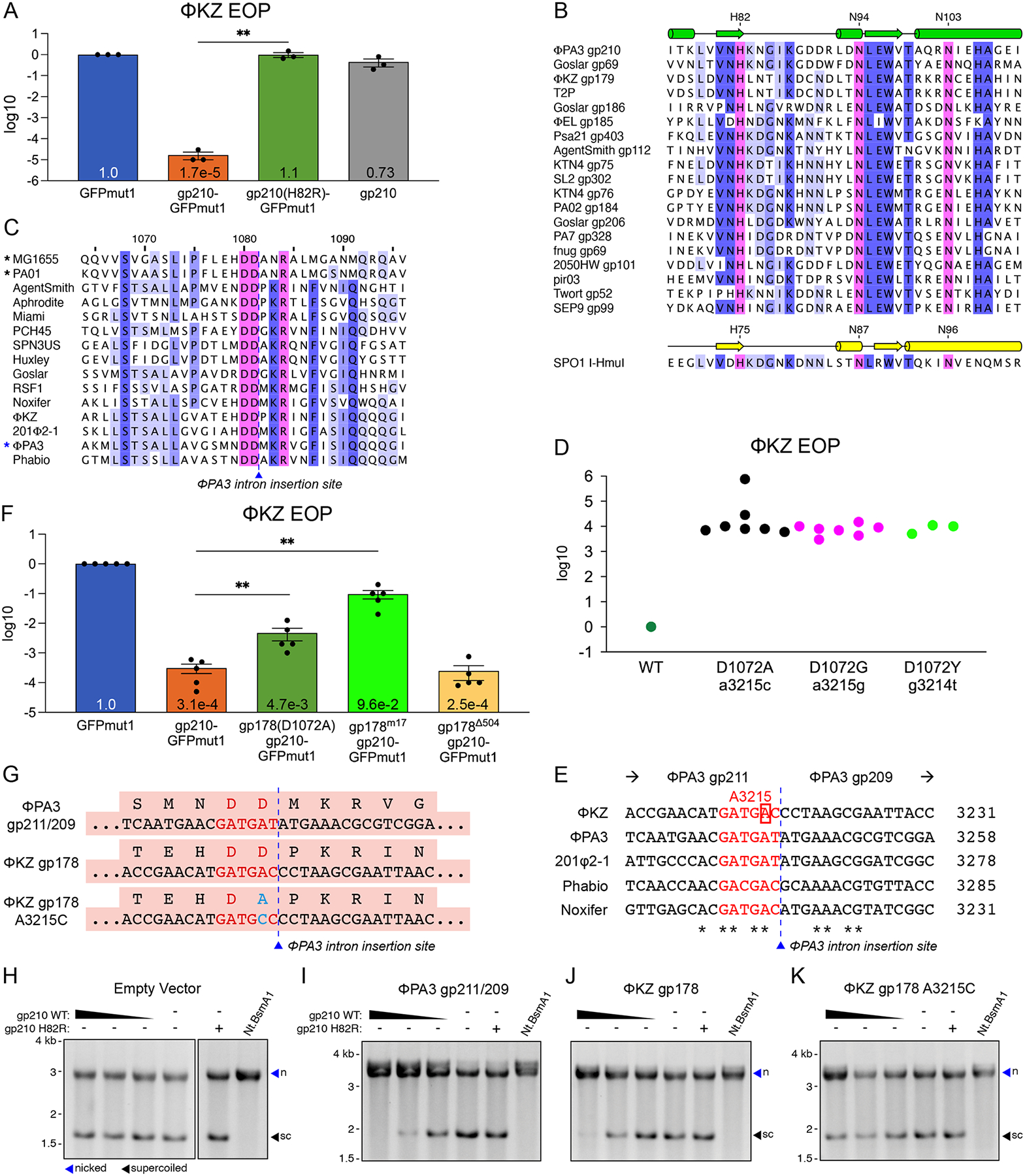
ΦPA3 gp210 is an HNH homing endonuclease that targets ΦKZ gp178. (A) Efficiency of plating (EOP) of ΦKZ as measured by spot titer and normalized to the paired titer on GFPmut1. gp210-GFPmut1 causes a 99.9983% decrease in ΦKZ titer (ratio paired t-test, p=0.0011, N=3) while untagged gp210, not imported into the nucleus, causes an insignificant 27% decrease (ratio paired t-test, p=0.14, N=3). H82R mutation (gp210(H82R)-GFPmut1) fully rescues ΦKZ titer compared to gp210-GFPmut1 (ratio paired t-test, **p=0.0026, N=3). Error bars represent SEM, p values were calculated by ratio paired t-test. (B) Protein alignment of phage-encoded endonucleases related to gp210 (top is predicted secondary structure from Phyre2), including well-characterized homing endonuclease I-HmuI (bottom with confirmed secondary structure). Cylinders represent α-helices and arrows indicate β-strands. Blue highlights indicate conservation at each position, and pink highlights indicate catalytic residues. ClustalO alignment. (C) Protein alignment including the intron(−) version of the ΦPA3 RNAP (blue asterisk), after editing from the annotation in Genbank which can be found in Figure S1D. It is aligned with RpoB of MG1655 (*E. coli*) and PAO1 (*P. aeruginosa*) (black asterisks), 8 RNAPs encoded by chimalliviruses infecting different genera of hosts, and 4 RNAPs encoded by other *Pseudomonas* chimalliviruses. Blue highlights indicate conservation at each position, and pink highlights indicate catalytic residues. ClustalO alignment. The intron insertion site occupied in the ΦPA3 gene is indicated. Residue numeration above is based on the ΦPA3 sequence. (D) Efficiency of plating (EOP) of ΦKZ escape mutants relative to the wildtype ΦKZ on cells expressing gp210-GFPmut1. (E) Nucleotide alignment of intron(−) RpoB homologs in five *Pseudomonas* jumbo phages. The point mutation in the ΦKZ vRNAP gp178 at position 3215 (red box) is two bases upstream of the site that aligns with the intron insertion site (black dotted line) in ΦPA3. Red sequences code for the conserved aspartic acid residues. The exons of ΦPA3 are noted above. (F) EOP of ΦKZ on cells expressing GFPmut1 as a control, gp210-GFPmut1, or co-expressing gp210-GFPmut1 and one of three ΦKZ gp178 gene variants. The nucleotide sequences of these variants are shown in Figure S2F. Gp178(D1072A) is the mutant found in escaper phages. Gp178^m17^ has 17 silent mutations that do not change amino acid sequence but are expected to interfere with gp210 nucleotide recognition. Gp178^Δ504^ has 504 nucleotides deleted (ratio paired t-test, **p=0.0034, N=5). (G) Sequences surrounding the insertion site of the intron containing gp210. ΦPA3 gp211/209: intron(−) version of the ΦPA3 vRNAP subunit. ΦKZ gp178: homologous vRNAP subunit. ΦKZ gp178 A3215C: gp178 with single nucleotide mutation. (H-K) Nuclease assay of purified gp210 incubated with plasmid DNA containing only the empty vector (H), the intron(−) ΦPA3 gene (I, gp211/209), ΦKZgp178 gene (J) or ΦKZgp178 gene with single nucleotide change A3215C (K). Gp210 concentration from left to right was 5.0, 2.5, 1.25, and 0 μM. Nt.BsaI is a reference digest for nicked plasmid (blue arrows, n). Supercoiled plasmid (sc) is indicated by black arrows.

Here we describe the discovery of a homing endonuclease, gp210, encoded within an intron in the genome of phage ΦPA3 and investigate its role in viral competition. Previously, we characterized intracellular speciation factors that separate the gene pools of co-infecting phages by studying ΦKZ and ΦPA3 of *Pseudomonas aeruginosa* (17), two members of the proposed *Chimalliviridae* family (18). These phages form a proteinaceous phage nucleus (19–21) that shields their DNA genomes from host defenses (22–24) and, like the eukaryotic nucleus, these phages can strictly separate transcription and DNA replication from translation and metabolic processes. Certain proteins are selectively imported into the phage nucleus, while others are excluded, and the selectivity of this process is determined by the conserved chimallivirus protein PicA (25). The ΦPA3-encoded endonuclease gp210 is naturally imported into ΦPA3’s phage nucleus, but is excluded from ΦKZ’s (17). To test the outcome of artificially importing gp210 into the ΦKZ nucleus, we tagged it with green fluorescent protein (GFP) variant GFPmut1, which is imported into the ΦKZ phage nucleus and can be fused to non-nuclear proteins to force their import (other GFP variants, such as sfGFP, are excluded from the phage nucleus of both ΦKZ and ΦPA3) (17, 22). Expressing gp210-GFPmut1 in host cells during infection with ΦKZ inhibited plaque formation, resulting in an 0.0017% efficiency of plating (EOP) compared with control cells expressing only GFPmut1 (Fig. 1A, ratio paired t-test, p=0.0011, N=3). Given that ΦPA3 and ΦKZ can co-infect the same cell and assemble a hybrid phage nucleus (17), we reasoned that ΦPA3 gp210 might interfere with ΦKZ reproduction during natural co-infections and provide a selective advantage to ΦPA3.

## Gp210 is an HNH endonuclease that inhibits ΦKZ replication

Gp210 is predicted to be a histidine-asparagine-histidine (HNH) endonuclease based on sequence alignments and AlphaFold structure predictions (Figs. 1B and S1, A–D). We mutated the predicted active site in gp210 from histidine to arginine (H82R) and found that the expression of gp210(H82R)-GFPmut1 in *P. aeruginosa* cells did not reduce ΦKZ titer (Fig. 1A), despite import into the ΦKZ nucleus (Fig. S1F). In liquid culture, monitoring bacterial growth curves in the presence of phage showed that gp210(H82R)-GFPmut1 had no significant effect on the ability of ΦKZ to inhibit the growth of host cells (Fig. S1E). In contrast, gp210-GFPmut1 reduced the susceptibility of the host cells to ΦKZ, requiring a 1,000-fold higher multiplicity of infection (MOI) to suppress bacterial growth in liquid culture. However, ΦPA3 suppression of host cell growth in liquid or on agar plates was unaffected by expression of either gp210-GFPmut1 or gp210(H82R)-GFPmut1 (Fig. S2, A–C). These results show that ΦKZ is strongly inhibited by the import of gp210-GFPmut1 into the ΦKZ nucleus and this depends upon the predicted HNH endonuclease motif.

Gp210 is encoded within an intron interrupting a highly conserved RNA polymerase gene. Homing endonucleases are often encoded within self-splicing group I introns that insert into a highly conserved region of an essential gene and they target the intron insertion site, as well as an homologous locus in co-infecting phages (26, 27). Gp210 is encoded within a putative group I self-splicing intron that interrupts the gene encoding a β subunit of one of the ΦPA3 RNA polymerases (RNAPs). It is inserted immediately after two highly conserved aspartic acid residues (Fig. 1C), the second of which has been implicated in fidelity of the RpoB RNA polymerase subunit in *E. coli* (D675) (28). To determine whether gp210 is a nuclease that can target the conserved region within the ΦKZ genome, we analyzed ΦKZ escaper mutants that infected *P. aeruginosa* expressing gp210-GFPmut1 (Fig. 1, D and E). We isolated fourteen spontaneous escaper mutants with a mutation of the adenosine at position 3215 of the ΦKZ gp178 gene which encodes the homologous β subunit of the RNAP subunit that is packaged into ΦKZ virions and transcribes early genes (29). The DNA sequence surrounding this site is highly conserved among chimalliviruses (Fig. 1E). This nucleotide change resulted in either a D1072A or D1072G amino acid change (Fig. 1D). A further three escapers mutated the guanine at position 3214, resulting in a ΦKZ gp178 D1072Y mutation. D1072 of ΦKZ gp178 aligns with the second aspartic acid at the end of ΦPA3 gp211, which borders the gp210 intron insertion site in ΦPA3 (Fig. 1, C and G). The overlap of fourteen escape mutations indicates site-specific targeting of the ΦKZ genome by gp210 and the proximity of this site to the intron insertion site in ΦPA3 is indicative of typical homing activity.

## Gp210 is a homing endonuclease that nicks the ΦKZ gp178 gene

To verify that gp210 is a homing endonuclease that targets the intron(−) version of its genomic locus in ΦPA3 and the homologous gene in ΦKZ, we created plasmids containing the uninterrupted sequence of the insertion site in the ΦPA3 vRNAP subunit (ΦPA3 gp211/209) or the matching locus in the homologous ΦKZ gp178 gene and tested the nucleolytic activity of purified gp210 *in vitro* (Figures 1H–K). While the empty vector was not cleaved by gp210, both the intron(−) ΦPA3 gp211/209 sequence and the ΦKZ gp178 sequence were nicked to convert supercoiled plasmid into an open circular structure, similar to the nicking control enzyme Nt.BsmA1 (Fig. 1, H-J). This activity required an intact HNH domain, as the H82R mutant protein showed no DNA nicking. Nicking by ΦPA3 gp210 of a modified ΦKZ gp178 sequence carrying the A3215C mutation was reduced (Fig. 1K). Together, these results show that ΦPA3 gp210 is capable of targeting the gene encoding the ΦKZ vRNAP subunit gp178.

The above results indicate that ΦPA3 gp210 nicks the ΦKZ genome within the gp178 gene, and that either the loss of this gene product or the lost integrity of the genomic DNA itself is responsible for the ΦPA3 gp210-dependent knockdown of ΦKZ viral titer. To distinguish between these hypotheses, we determined whether expression of gp178 on a plasmid can rescue ΦKZ replication from gp210-GFPmut1. We co-expressed gp210-GFPmut1 with either gp178(D1072A) containing adenosine 3215 mutated to cytosine or gp178 with seventeen silent mutations near the putative gp210 target site to prevent gp210 recognition but conserve the amino acid sequence (gp178^m17^) (Fig. S2F). We found that co-expression of gp178(D1072A) resulted in a 15-fold increase in ΦKZ titer compared to gp210-GFPmut1 alone (ratio paired t-test, p=0.0034, N=5). Co-expression of gp178^m17^ resulted in a 300-fold increase in titer compared with gp210-GFPmut1 alone (Fig. 1F, ratio paired t-test, p=0.0012, N=5). Co-expression of gp178 with a large internal in-frame deletion of 504 bases (gp178^Δ504^) did not rescue ΦKZ (ratio paired t-test, p=0.66, N=5), showing that inserting a gene upstream of gp210 was not responsible for preventing gp210 activity. Consistent with the ΦKZ gp178 gene containing the recognition sequence for gp210, we could not construct a plasmid for co-expression of gp210-GFPmut1 and the wildtype gp178 gene sequence. Expression of a wildtype gp178 protein from a gene that has been recoded to avoid recognition by gp210 restored phage viability, demonstrating that the loss of phage titer is due to the absence of gp178..

## Gp210 prevents production of viable ΦKZ virions

**Figure 2.**
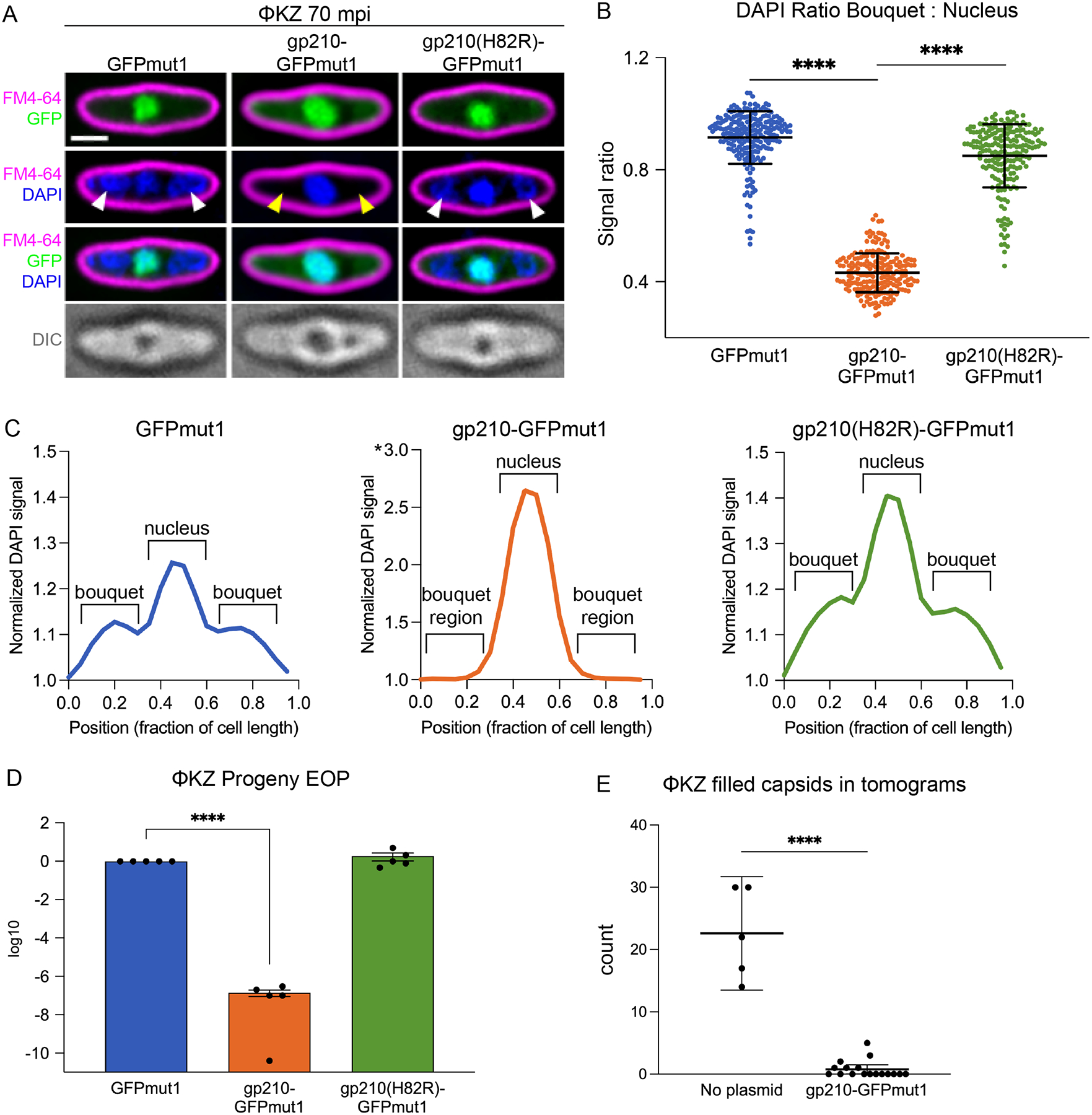
Gp210-GFPmut1 causes a loss of ΦKZ progeny. (A) Live fluorescence microscopy of ΦKZ infections stained with FM4–64 (magenta: membranes) and DAPI (blue: DNA) 70 minutes post infection (mpi). Infections proceeded in the presence of either GFPmut1, gp210-GFPmut1, or gp210(H82R)-GFPmut1 (green: GFP). DAPI stained capsids in phage bouquets are labeled by white arrowheads. Absence of DAPI stained capsids in cells expressing gp210-GFPmut1 noted with yellow arrowheads. DIC: Differential Interference Contrast. Scale bar is 1 μm. (B) Ratio of DAPI signal in the bouquet compared to the nucleus at 70 mpi. Unpaired t-test, ****p<0.0001. Error bars represent standard deviation. GFPmut1 n=220, gp210-GFPmut1 and gp210(H82R)-GFPmut1 n=200. (C) Line plots of DAPI signal intensity along a bisecting line at 70 mpi, average of n=50. * note that the y-axis for gp210-GFPmut1 is double that of the other panels. (D) EOP of ΦKZ progeny (logscale) collected from washed infections of cells expressing the indicated protein, measured by spot titer on cells without plasmid. Progeny grown in the presence of gp210-GFPmut1 plaque with an efficiency of 0.00001% of the progeny grown with GFPmut1 (ratio paired t-test, p=0.0005, N=5). Progeny produced with gp210(H82R)-GFPmut1 have a relative EOP of 190% (ratio paired t-test, p=0.56, N=5). Error bars represent SEM. (E) Number of capsids filled with DNA observed in tomograms of the control strain (no plasmid, N=5) or the strain expressing gp210-GFPmut1 (N=17) at 90 mpi. Unpaired t-test, ****p<0.0001.

Despite the inhibition of ΦKZ titer in cells expressing gp210-GFPmut1, the phage nucleus still formed, enlarged, and was centered at mid-cell as observed by bright DAPI (4′,6-diamidino-2-phenylindole) staining of the viral DNA (Fig. 2A). Normally at 70 minutes post infection (mpi), ΦKZ particles containing DNA assemble into bouquet structures that can be visualized with DAPI staining (30). However, expression of gp210-GFPmut1 resulted in a lack of stained capsids in the ΦKZ phage bouquets by 70 mpi (Fig. 2A, yellow arrowheads). This change in the distribution of DAPI staining was measured by line plots of DAPI intensity along a line bisecting the long axis of the cell (Fig. 2C, N=50), supporting the conclusion that in strains expressing gp210-GFPmut1, ΦKZ capsids containing viral DNA do not accumulate while nuclear DNA increases approximately two-fold. The ratio of DAPI staining in the nucleus compared to the cytosolic regions on either side of the nucleus that typically contain bouquets revealed a significant decrease (unpaired t-test, p<0.0001, N≥200) in bouquet staining relative to nuclear staining when gp210-GFPmut1 was expressed, compared with GFPmut1 alone or mutant gp210(H82R)-GFPmut1 (Fig. 2B). In contrast, ΦPA3 infection morphology and bouquet formation was undisturbed by the expression of gp210 (Fig. S2B).

To test if viable ΦKZ progeny were produced from infections containing gp210-GFPmut1, cells were infected with ΦKZ, washed to remove unbound parent phage, and the host cells were allowed to lyse and release the progeny for collection after 2 hours. The ΦKZ progeny lysate produced in cells expressing gp210-GFPmut1 plated with an efficiency of 0.000014% (ratio paired t-test, p=0.0005, N=5) compared with the progeny produced in cells expressing GFPmut1 alone. This reduction of viable progeny was not observed in cells expressing gp210(H82R)-GFPmut1 (Fig. 2D). Together, these results indicate that gp210-GFPmut1 prevents the accumulation of ΦKZ capsids with DNA and thus the production of infectious virions.

**Figure 3.**
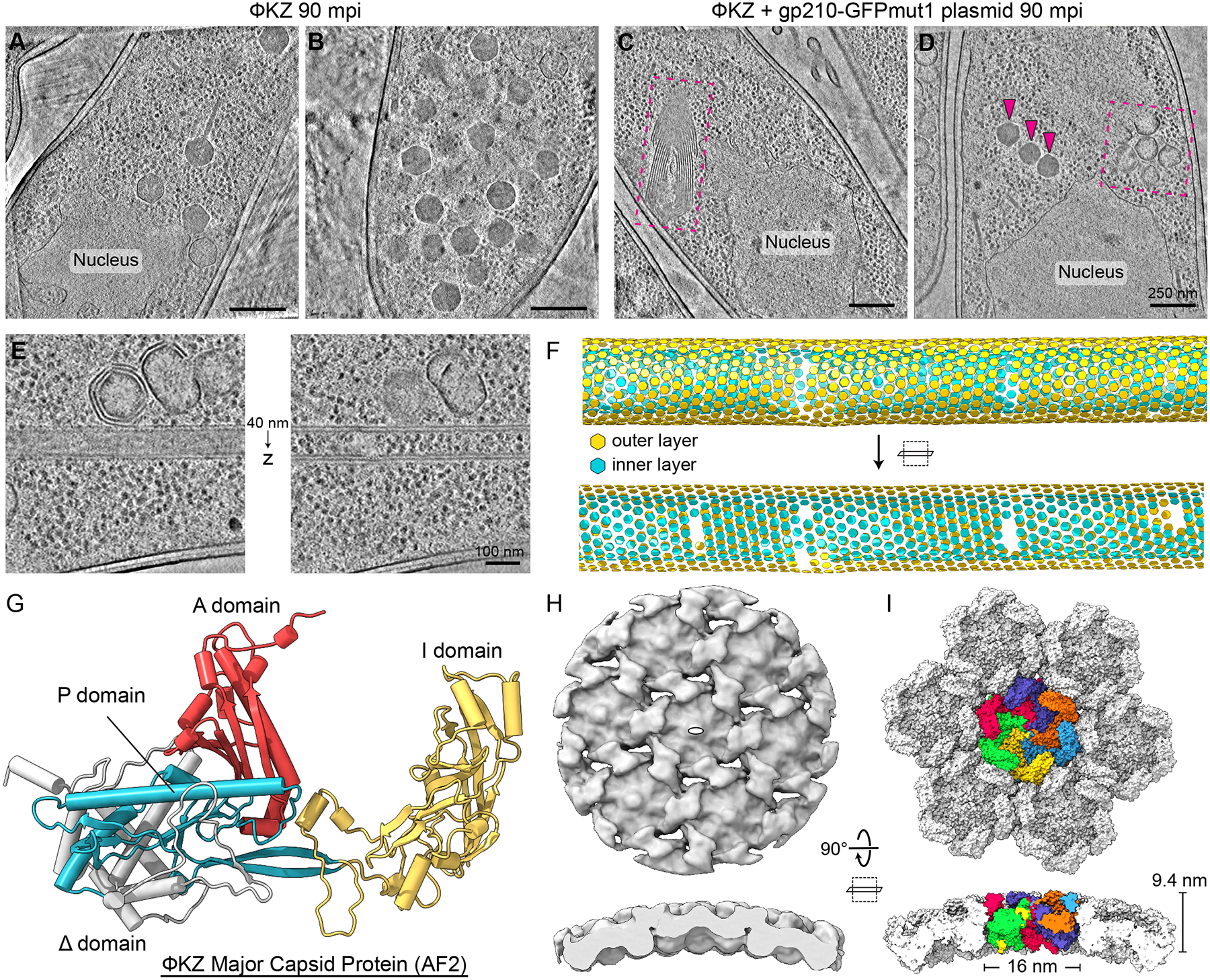
Targeting of ΦPA3 gp210 to ΦKZ phage nucleus results in misassembly of ΦKZ major capsid protein. (A, B) Cryo-FIB-ET of ΦKZ-infected *P. aeruginosa* cells at 90 mpi. (C, D) Cryo-FIB-ET of ΦKZ-infected *P. aeruginosa* cells expressing ΦPA3 gp210-GFPmut1 at 90 mpi. Magenta arrows point to a few properly assembled ΦKZ capsids and magenta dashed boxes indicate regions containing misassembled capsomer complexes. (E) Tomogram slices of a double-layered capsomer tube separated by 40 nm in the Z-direction. (F) Lattice plot of aligned subtomograms from the region depicted in (E). Subtomograms from the outer and inner layers are colored yellow and cyan, respectively. (G) AlphaFold2/ColabFold predicted structure of the ΦKZ major capsid protein (MCP) depicted as a cartoon model. The structure is annotated according to the HK97-fold features. (H) Exterior face and slabbed side views of the subtomogram reconstruction of the ΦKZ MCP from the tubular assemblies with a white oval indicating the 2-fold symmetry. (I) Same views as in (H) of the surface representation of the fitted model of the ΦKZ MCP protomer into the subtomogram reconstruction with the central hexamer protomers colored individually and surrounding hexamers colored white. Scale bars A-D: 250 nm, E: 100 nm.

The loss of infective progeny virions (Fig. 2D) and the lack of DAPI-stained phage bouquets (Fig. 2, A-C) led us to hypothesize that gp210 nicking of the ΦKZ gp178 gene disrupts virion production. To visualize the macromolecular organization of a ΦKZ infection in the presence of gp210-GFPmut1, we performed cryo-focused ion beam milling coupled with cryo-electron tomography (cryo-FIB-ET). ΦKZ-infected *P. aeruginosa* lacking plasmid contained both empty capsids and capsids full of DNA, with an average of 22.6 full capsids per tomogram (Fig. 2E, ±3.3 SEM, N=5) that were grouped into bouquets by 90 mpi (Figs. 3, A-B and S3C). However, when gp210-GFPmut1 was expressed (Fig. 3, C and D), only a few filled capsids were observed (Fig. 3D, magenta arrows), with an average of 0.76 full capsids per tomogram (Fig. 2E, ± 0.34 SEM, N=17), showing a 96.6% decrease in virion production (Fig. 2E, unpaired t-test, p<0.0001) in agreement with our fluorescence microscopy results (Fig. 2, A-C).

Cryo-FIB-ET of ΦKZ-infected cells expressing gp210-GFPmut1 revealed a significant deficit of filled capsids in addition to many other unusual structures (Fig. 3, C and D, magenta dashed boxes). Some assemblies conformed to capsid-like geometries, while others were tubular structures of variable diameter (Fig. 3, E and F). Regardless of shape, the layers of all these structures possessed a similar texture and thickness of ~9.4 nm (Fig. 3, H and I), suggesting a common elementary unit. Through subtomogram analysis of the tubular structures, we obtained an approximately 13 Å resolution map on which we could fit a predicted structure of the immature ΦKZ major capsid protein (MCP, gp120) to explain the density (Figs. 3, E-I and S4). The immature status of the MCP is marked by the presence of the N-terminal Δ-domain, which is proteolytically cleaved upon proper capsid maturation (31) (Figs. 3G and S5). Previous work on the MCPs of phages P22 and T4 described similar types of geometries (e.g., irregularly sized capsids, spiral lattices, and tubular “polyheads”) when the initial capsid nucleation and scaffolding process was disrupted (32, 33). These results show that gp210 targeting of the ΦKZ gp178 gene disrupts capsid assembly.

## Gp210 confers a competitive advantage over co-infecting phage

**Figure 4.**
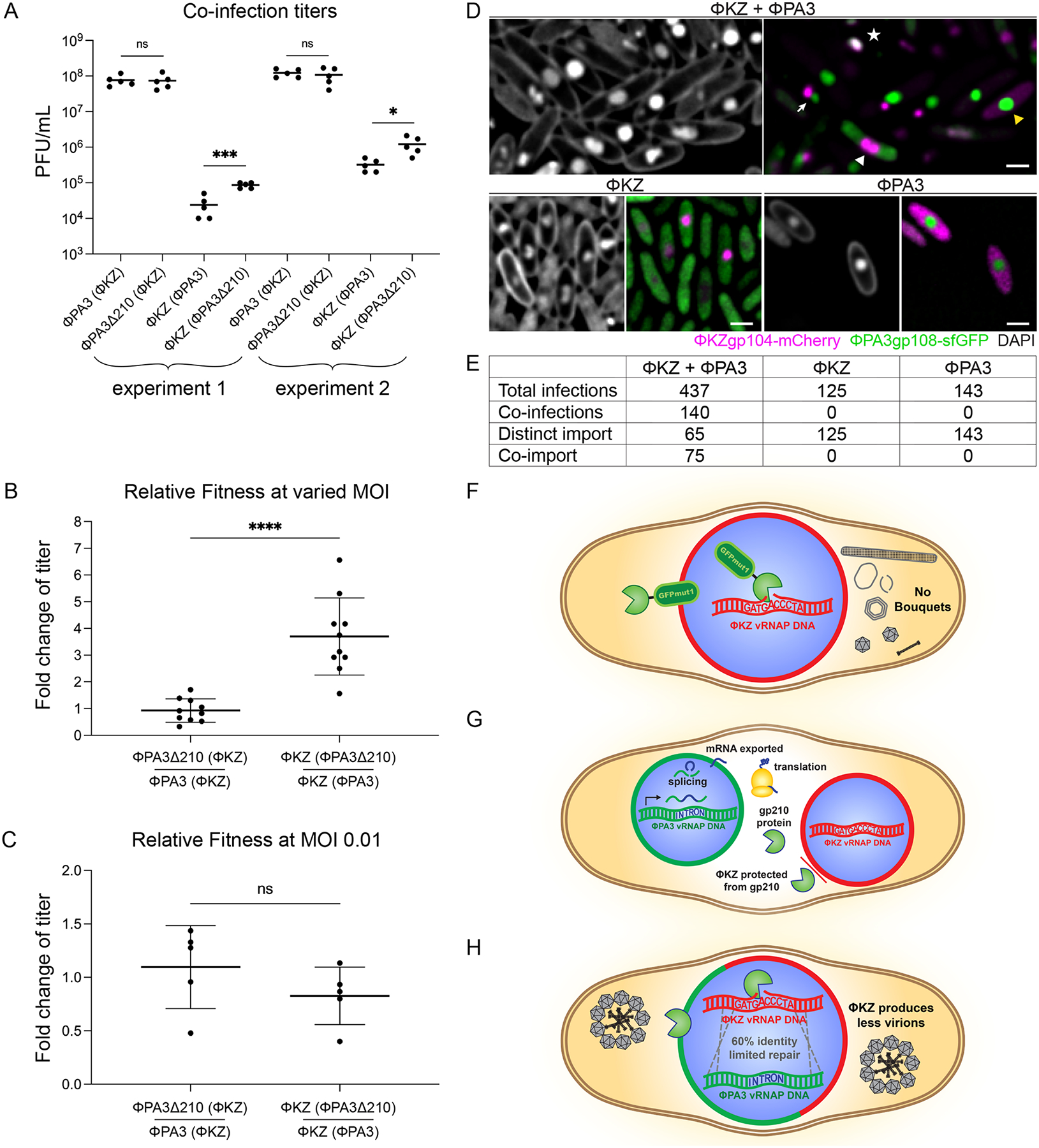
Gp210 provides a competitive advantage in phage co-infections. (A) Titer (PFU/ml) of ΦPA3 (starting MOI 10) or ΦKZ (starting MOI 0.1) after one round of competition with either wildtype ΦPA3 (ΦPA3) or ΦPA3 lacking gp210 (ΦPA3Δ210; Figure S6). Two experiments were conducted with 5 independent replicates each. ΦKZ has significantly higher titers after one round of competition with ΦPA3Δ210 compared to wildtype ΦPA3 (unpaired t-test, *p=0.014, ***p=0.0003). (B) Fold change of titers from (A) of ΦPA3Δ210 relative to ΦPA3 when in competition with ΦKZ (left) or ΦKZ in competition with ΦPA3Δ210 compared to ΦKZ in competition with ΦPA3 (right) (unpaired t-test, ****p<0.0001). Fold-change was determined by dividing each of the experimental titers (ΦPA3Δ210(ΦKZ), ΦKZ(ΦPA3Δ210)) by the mean of the control titers (ΦPA3(ΦKZ), ΦKZ(ΦPA3)) for each experiment. (C) Fold change of titers of ΦPA3Δ210 relative to ΦPA3 when in competition with ΦKZ (left) or ΦKZ in competition with ΦPA3Δ210 compared to ΦKZ in competition with ΦPA3 (right) when both phage are present at an MOI of 0.01 (unpaired t-test, p=0.24). Fold-change was determined by dividing each of the experimental titers (ΦPA3Δ210(ΦKZ), ΦKZ(ΦPA3Δ210)) by the mean of the control titers (ΦPA3(ΦKZ), ΦKZ(ΦPA3)) for each experiment. (D) ΦKZgp104-mCherry (magenta) and ΦPA3gp108-sfGFP (green) were co-expressed in *P. aeruginosa* and infected with both ΦKZ and ΦPA3 (top panels: DAPI staining left, right mCherry and sfGFP right), ΦKZ alone (bottom panels, DAPI left, mCherry and sfGFP right)), or ΦPA3 alone (bottom panels, DAPI left, mCherry and sfGFP right). In the top right image, an example of a phage nucleus importing both proteins (co-import) is indicated by a white star, a co-infection with two separate nuclei importing either the ΦKZ or the ΦPA3 protein is shown by a white arrow, two nuclei importing only the ΦKZ protein is marked by a white arrowhead, and a nucleus importing only the ΦPA3 protein is pointed out by a yellow arrowhead. DIC is shown in gray. Scale bars are 1 μm. (E) Quantitation of fields from the experiment in (D). “Co-infections” is when import of both proteins into the same or distinct nuclei is observed, “Distinct import” is when the nuclear proteins were not colocalized, and “Co-import” is when both proteins were colocalized in the same nucleus. (F) gp210 (green pacman) tagged with GFPmut1 is artificially imported into the ΦKZ nucleus (red shell, blue fill) where it cuts ΦKZ DNA in the vRNAP gene of gp178, dependent upon adenosine 3215, inhibiting ΦKZ bouquet formation. (G) When separate nuclei are formed by ΦKZ (red) and ΦPA3 (green), ΦPA3 expresses gp210 which is translated in the cytosol and encounters the ΦKZ nuclear shell. ΦKZ excludes gp210 allowing it to replicate simultaneously with ΦPA3. (H) Hypothesis of the effects of gp210 on a hybrid nucleus; ~50% of co-infections (E). In a hybrid nucleus, gp210 would be imported and cut the ΦKZ vRNAP gene gp178. Recombination efficiency is reduced with ~60% identity between the vRNAP alleles, leading to a competitive advantage for ΦPA3.

This work shows that gp210 is a homing endonuclease encoded within an intron interrupting a ΦPA3 vRNAP gene. If gp210 is imported into the ΦKZ nucleus, it is able to cut the vRNAP gene at the site homologous to the intron insertion site in ΦPA3 (Fig. 4F). This results in the inhibition of ΦKZ virion assembly and prevents subsequent rounds of infection, significantly reducing ΦKZ fitness. Given the observed effects on ΦKZ fitness, we asked whether this intron containing the gp210 homing endonuclease provides an advantage to phage ΦPA3 during competition with ΦKZ. Although gp210 is not naturally imported into the ΦKZ nucleus, it might be imported into mixed phage nuclei during competition. We created an isogenic ΦPA3 lysate lacking gp210 (ΦPA3Δ210) using Cas13a (34) (Fig. S6) and quantified the outcome of ΦPA3/ΦKZ competition after a single round of co-infection. Cells were infected with an MOI of 10 for ΦPA3 and of 0.1 for ΦKZ, such that most cells infected by ΦKZ would be co-infected with ΦPA3. Cells were washed to remove unbound phage and the number of ΦKZ progeny was determined after one replication cycle by plating on cells that ΦPA3 could not infect. We found that the ΦKZ population size was 3.7-fold less when competing against wildtype ΦPA3 with gp210 compared to mutant ΦPA3 lacking gp210 (unpaired t-test, p<0.0001, N=10), while wildtype and mutant ΦPA3 produced equivalent numbers of viable progeny (Fig. 4, A and B). This 3.7-fold difference provides an advantage to ΦPA3 that would be missing without gp210 and shows the adaptive benefit of homing endonucleases for viral genomes. Gp210 does not give ΦPA3 a direct replicative advantage as ΦPA3Δ210 produced approximately equal progeny as wildtype ΦPA3 during single cycle competition with ΦKZ (Fig. 4, A and B). However, by inhibiting ΦKZ replication (Figs. 1–3) and thereby reducing the number of ΦKZ virions in the population that are competing for host cells, gp210 provides a relative fitness advantage to ΦPA3 compared to ΦPA3Δ210. When wildtype ΦPA3 or ΦPA3Δ210 competed against ΦKZ at low MOIs where co-infections are minimized, the fitness advantage of gp210 disappeared (Fig. 4C). This demonstrates that the relative fitness advantage imparted on ΦPA3 compared to ΦPA3Δ210 is dependent on intracellular co-infection with ΦKZ.

This fitness advantage is predicted to rely upon the ability of ΦKZ to import ΦPA3 proteins when mixed nuclei are formed during co-infection. To determine if ΦKZ and ΦPA3 are able to import each other’s proteins during co-infection, we co-expressed ΦKZgp104-mCherry and ΦPA3gp108-sfGFP. These two related proteins are only imported by their cognate phage during infections with either ΦKZ or ΦPA3 (Fig. 4, D and E, “ΦPA3” and “ΦKZ”) (25). However, during co-infections, 46.4% (N=140) of nuclei are formed that only import one protein, while the other 53.6% import both proteins (Fig. 4, D and E, “ΦKZ + ΦPA3”). This data shows that hybrid nuclei are formed during co-infection that are capable of importing proteins from both ΦPA3 and ΦKZ. While previous quantification of viral DNA based on DAPI staining (15) suggests that ΦKZ is able to replicate faster than ΦPA3, potentially giving ΦKZ a replicative advantage, mathematical modeling shows that gp210 helps the less competitive phage ΦPA3 during co-infections with ΦKZ (Figs. S8–S10).

These results together with our molecular characterization of the effect of gp210 on ΦKZ replication support a mechanism by which ΦKZ fitness is reduced in the presence of ΦPA3 expressing gp210. Thus, gp210 provides a relative fitness advantage to its host ΦPA3 when challenged by co-infection with ΦKZ.

## Discussion and limitations

Introns are widespread, but how often or under what circumstances do they evolve into interfering agents capable of being deployed during viral competition? Here we have identified a viral homing endonuclease that provides a competitive advantage to the host by killing its competitor rather than acting as a purely selfish genetic element. The gp210 endonuclease encoded by ΦPA3 is excluded from the ΦKZ nucleus (Fig. 4D) but it is expected to be imported into hybrid nuclei formed by both phage during co-infection (Fig. 4, D, E, and model H) (17). Within these hybrid nuclei, recombination repair using the ΦPA3 genome is likely to be limited by the amount of divergence between the genomes, preventing ΦKZ from gaining protection from gp210 by acquiring the intron, explaining the observed competitive advantage. Overall, our results demonstrate that viruses containing intron endonucleases can have a strong fitness advantage during competition and provide a possible explanation for the ubiquity of these genetic elements among viruses.

The condition for homing endonuclease-mediated interference is likely not limited to the formation of hybrid nuclei. Hypothetically, for newly diverging chimalliviruses that still share nuclear import pathways, such as the ancestors of ΦPA3 and ΦKZ, acquisition of an intron encoding a homing endonuclease by one phage would confer a strong competitive advantage. During co-infection of these related phages, import of a homing endonuclease into both nuclei results in a cut in the genome of the phage lacking the intron, which cannot be repaired by the intron-containing genome that is sequestered in the other nucleus. This makes the endonuclease a potent weapon that inhibits closely related co-infecting phages lacking the intron, and also drives the evolutionary divergence of the phage nucleus to exclude these toxic proteins, as is the case for ΦKZ.

The general mechanism of homing endonucleases is conserved and they are widespread among viruses. Therefore, it is likely that they endow other viruses with a similar competitive advantage over co-infecting relatives. Since homing endonucleases in other phages target genes such as thymidylate synthase and DNA polymerase (35–38), the phenotype of this targeting may differ depending on which gene is inactivated. Mechanisms that result in the spatial separation of viral genomes occur in eukaryotic as well as bacterial cells (15, 39–41) and may provide the conditions for homing endonuclease-mediated interference competition due to reduced recombination with the intron(+) genome needed to repair the cut made by the nuclease (Fig. S7C). For co-infecting viral genomes that replicate in close proximity without a physical barrier, the intron will spread if there is sufficient homology (Fig. S7A) but recombination can be limited by sequence divergence (42) (Fig. S7B). Mobile introns are known to prefer highly conserved regions of essential genes (43) so, as two genomes diverge, the nuclease target site remains largely unchanged while the surrounding DNA loses homology with the competitor, limiting rescue by homologous recombination. In this way, the homing endonuclease of the mobile intron can be deployed against related but divergent phages, even without subcellular genetic isolation.

One limitation of this work is that it studies phage competition during a single round of infection. We expect that future studies including long-term passaging experiments will show the effects of a homing endonuclease in intracellular competition dynamics over longer evolutionary time scales. Another limitation is that we are unable at this time to engineer specific mutations into the phage genome, thus we can show a fitness penalty for loss-of-function mutations, but not a fitness increase from gain-of-function insertions.

## Conclusion

Homing endonucleases are broadly distributed across diverse phage families as well as in fungi and archaea (15, 44–47) and they have now been shown to have the potential to facilitate intracellular interference competition. This mechanism is especially important in the evolutionary arms race between viruses because of constant competition through co-infection (48) and rapid rates of viral replication that quickly amplify even small selective advantages. Figure 1B displays a small sampling of highly conserved gp210-related endonucleases found in phages, which infect a wide variety of host cells, including Gram negative and Gram positive bacteria isolated from many environments (18). Every homing endonuclease has the potential to evolve as a weapon, owing to the differences in rates of divergence between conserved target sites and the surrounding genome (42). In addition, regardless of sequence divergence, any conditions that physically separate two intracellular genomes but allow mixing of their encoded proteins present the potential to involve homing endonuclease-mediated interference competition if one of the genomes acquires a homing endonuclease. The relative fitness advantage we observed that is provided by a weaponized homing endonuclease could influence any intracellular genetic competition, including those arising between plasmids (49), viruses, and hosts.

## Supplementary Material

Supplement

## Data Availability

The final subtomogram average of the ΦKZ major capsid protein from this study has been deposited with the Electron Microscopy Data Bank with accession number EMD-40674. The Alphafold2 generated coordinate model was deposited with Zenodo(50).
