# Supplementary material for: An intron endonuclease facilitates interference competition between co-infecting viruses
Source: Science. Author manuscript; Available in PMC 2024 Dec 5. (PMC11620839; doi:10.1126/science.adl1356)
Supplement: Supplement [file NIHMS2034457-supplement-Supplement.pdf]

Science
AAAS

Supplementary Materials for

A mobile intron facilitates interference competition between co-infecting viruses

Erica A. Birkholz[1,5], Chase J. Morgan[1,5], Thomas G. Laughlin[1], Rebecca K. Lau[3], Amy Prichard[1], Sahana Rangarajan[1], Gabrielle N. Meza[1], Jina Lee[1], Emily Armbruster[1], Sergey Suslov[1], Kit Pogliano[1], Justin R. Meyer[2], Elizabeth Villa[1,4], Kevin D. Corbett[1,3], Joe Pogliano[1]‡

Correspondence to: jpogliano@ucsd.edu

This PDF file includes:

Materials and Methods
Figs. S1 to S10

Materials and Methods

Bacterial strains, phage, and growth conditions

P. aeruginosa PAO1 derivative K2733 was used as the host in all experiments. It was grown at 30°C in LB and 15 µg/ml gentamycin was used for plasmid selection. Phage stocks of ΦKZ and ΦPA3 were collected from medium titer plate lysates using phage buffer (10 mM Tris (pH 7.5), 10 mM $MgSO_4$, 68 mM NaCl, and 1 mM $CaCl_2$) and stored at 4°C with titers of 10^{12} and 10^{10}, respectively.

Plasmid construction and transformation

Plasmids were either constructed by Gibson assembly (*15*) or synthesized and cloned by Genscript (see Table S2). The vector for all plasmids was pHERD-30T (*15*, *17*, *18*, *48*) and Genscript cloned the inserts using SacI and SalI. Plasmid transformation was accomplished by 2 kV electroporation of K2733 cells washed with 300mM sucrose and stored at -80°C.

Generation of gp210-Deficient Mutant ΦPA3

P. aeruginosa K2733 cells containing Cas13a expression vector pHERD30T_LbuCas13a_PhiPA3gp210_HNN_Asn1g1 with guide targeting the HNN endonuclease active site of gp210 (TCTAAGTTATCAAGGCGGTCATCGCCTTTTA) were grown to OD= ~0.5 in LB containing 15 µg/mL gentamicin and 1% arabinose. 100 µL of culture were infected with ~10^9 PFU of ΦPA3 and mixed with 5mL of LB 0.2% arabinose 0.35% agar cooled to 55°C. Lawns of bacteria expressing gp210-targeting Cas13a were grown by plating this mixture on LB 15 µg/mL gentamicin 1.5% agar. Individual plaques containing escape mutants were selected and streak-purified three more times on lawns of bacteria expressing gp210-targeting Cas13a. The region of the genome targeted by Cas13a was then amplified by PCR and the amplicon sequenced by Sanger sequencing to determine mutation.

ΦPA3 ΦKZ Co-infection and Quantification

P. aeruginosa K2733 was grown in LB and diluted to an OD 0.1. 1 mL of culture was infected with ~10^9 PFU of either ΦPA3 or ΦPA3Δ210 (MOI: ~10) and ~10^7 PFU of ΦKZ (MOI:~0.1). Infected cultures were incubated for 5 min at 30°C rotating and then the cells were pelleted at 8,000rcf for 1.5 min. Pellets were washed with 1mL of LB three times and then infected cells were resuspended in 200uL of LB and incubated while rotating at 30°C for 90 minutes (~1 infection cycle). Remaining cells were pelleted and supernatant containing phage was removed and serially diluted from 10^{-1} to 10^{-8}. To distinguish ΦKZ from ΦPA3 in a mixed lysate for the experiments with ΦPA3 MOI =10, ΦKZ MOI=0.1, total phage concentration was determined by spot titer on lawns of K2733. Since ΦPA3 is in excess of ΦKZ by several orders of magnitude, the titer of phage on K2733 is equivalent to the titer of ΦPA3. ΦKZ concentration was determined by spot titer and whole plate titer on lawns of K2733 expressing Cas13a targeting the start codon of ΦPA3 chimallin (pHERD30T_Lbu_PhiPA3-shellg1, TTTGGGTCCTTGTTGGGTCTGTTGCATTTGC). Since ΦPA3 cannot replicate on the Cas13 strain targeting ΦPA3 chimallin, the titer only reflects the ΦKZ titer. When the competition was performed with both phage at MOI 0.01, ΦPA3 concentration was determined by spot titer on *P. aeruginosa* strain PA14, which is naturally resistant to ΦKZ. ΦKZ concentration was determined by spot titer and whole plate titer on lawns of K2733 expressing Cas13a targeting the start codon of ΦPA3 chimallin (pHERD30T_Lbu_PhiPA3-shellg1,

TTTGGGTCCTTGTTGGGTCTGTTGCATTTGC). Replicate titers from each experiment were normalized to the mean of the control condition (wildtype ΦPA3, ΦKZ co-infection) from each individual experiment and then combined to determine the mean fold-change in titer between the two conditions.

Protein Expression, Purification, and Characterization

Full length WT gp210 and mutant genes were cloned into UC Berkeley Macrolab vector 1B (Addgene #29653) to generate N-terminal fusions to a TEV protease-cleavable His_6-tag. Proteins were expressed in *E. coli* strain Rosetta2 pLysS (EMD Millipore) by induction with 0.25 mM IPTG at 20°C for 16 hours.

For protein purification, cells were harvested by centrifugation, suspended in resuspension buffer (20 mM Tris-HCl pH 7.5, 300 mM NaCl, 5 mM imidazole, 5 mM 2-mercaptoethanol and 10% glycerol) and lysed by sonication. Lysates were clarified by centrifugation (16,000 rpm 30 min), then supernatant was loaded onto a 5 mL Ni^{2+} affinity column (HisTrap HP, GE Life Sciences) pre-equilibrated with resuspension buffer. The column was washed with buffer containing 20 mM imidazole and 100 mM NaCl, and eluted with a buffer containing 250 mM imidazole and 100 mM NaCl. The elution was loaded onto an anion-exchange column (Hitrap Q HP, GE Life Sciences) and eluted using a 100-600 mM NaCl gradient. Fractions containing the protein were pooled and mixed with TEV protease (1:20 protease:gp210 by weight), then incubated 48 hours at 4°C for tag cleavage. Cleavage reactions were passed over a Ni^{2+} affinity column, and the flow-through containing cleaved protein was collected and concentrated to 2 mL by ultrafiltration (Amicon Ultra-15, EMD Millipore), then passed over a size exclusion column (HiLoad Superdex 200 PG, GE Life Sciences) in a buffer containing 20 mM Tris-HCl pH 7.5, 300 mM NaCl, and 1 mM dithiothreitol (DTT). Purified proteins were concentrated by ultrafiltration and aliquoted and frozen at -80°C for biochemical assays. All mutant proteins were purified as wild-type.

Nuclease activity assays

Plasmids were miniprepped from 5 mL of overnight culture of NovaBlue *E. coli* cells. Purified gp210 was mixed with 500 ng plasmid DNA in a buffer containing 10 mM Tris-HCl (pH 7.5), 25 mM NaCl, 10 mM $MgCl_2$, and 1 mM DTT (50 mL reaction volume), incubated 1 hour at 37°C, then separated on a 1.0% agarose gel. Gels were stained with ethidium bromide and imaged by UV illumination.

Efficiency of Plating (EOP) by spot titers

To determine the efficiency of phage plaque formation on bacteria expressing different fusion proteins, previously published methods were followed (*15*). Protein expression was achieved with the indicated fusions by inducing with 1.0% arabinose. For the EOP of progeny, the indicated expression protein was expressed during the replication of the progeny and the titers were performed on the *P. aeruginosa* host, K2733, without any plasmid present.

IC50 growth curves

Growth curves to determine the amount of phage required for suppression of the bacterial culture to 50% of maximum growth (IC50) were performed as described previously (*15*). 95 µl of bacterial host at OD_{600} of 0.1 was combined with 5 µl of ten-fold serial dilutions of a ΦKZ lysate across a 96-well plate. The plates were shaken for an initial 40 minutes at 30 °C in a

microplate reader (Tecan Infinite MPlex and Tecan Sunrise) after which OD_{600} measurements were taken every 10 minutes, with continuous shaking at 30 °C between the timepoints. The OD_{600} values were averaged and plotted as a growth curve. IC50 is annotated at half of the OD_{600} reached by the strain when grown with only phage buffer. Fusion proteins were expressed by 0.1% arabinose.

Single cell infection assay

Single cell infections of *P. aeruginosa* were visualized using fluorescence microscopy (*15*, *17*, *18*, *48*). Briefly, 1% agarose pads containing 25% LB, 2 µg/ml FM 4-64 (*49*), and 1 µg/ml DAPI (4′,6-diamidino-2-phenylindole) were inoculated with 5 µl of cells (OD_{600} 0.4) and incubated for 3 hours at 30°C in a humidor and then infected with 10 µl of a high titer phage lysate. Imaging was performed with a DeltaVision Elite Deconvolution microscope (Applied Precision, Issaquah, WA, USA). Images were further processed by the aggressive deconvolution algorithm in DeltaVision SoftWoRx 6.5.2 Image Analysis Program, and analyzed using Fiji 1.53c software.

Lysis time-lapse

Time-lapse to measure lysis was performed as previously described (*15*). Infections were established on agarose pads (described above) without any stains. Beginning after 45 minutes post-infection (mpi), images were captured every 10 minutes for 4 hours using UltimateFocus. The cells that lysed during the imaging were counted and reported as a percentage of the total number of starting cells. The percentages were averaged and plotted for both bacterial host strains.

Progeny collection

ΦKZ progeny were generated in the presence or absence of functional or nonfunctional ΦPA3gp210. Homogeneous liquid cultures of *P. aeruginosa* K2733 were grown in LB for 3 hours at 30°C. The OD_{600} was measured and cultures were back-diluted to an OD_{600} of 0.05 and induced with 1% arabinose for 1 hour to express the protein of interest (GFPmut1, gp210-GFPmut1, gp210-sfGFP, or gp210H82A-GFPmut1). Cells were grown to an OD_{600} of about 0.1 and infected with ΦKZ at an MOI of 30 to ensure high infection rates. During early infection (30 mpi, after cells would be infected but before lysis begins), cells were centrifuged at 3220 rcf for 10 minutes to pellet infected cells and the supernatant was removed, filtered through a 0.45 µm filter to remove any residual bacterial cells, and saved as the "parent" phage sample. Cells were resuspended in fresh LB and washed twice more by centrifugation and resuspension to remove any residual parent phage from the supernatant. When the infections would be entering later stages (80 mpi), centrifugation was halted to prevent premature lysis of infected cells and resuspended cells were concentrated 5-fold in LB to ensure a high titer of progeny phage and incubated for 2-4 hours at 30°C to allow for complete lysis of infected cells. After incubation, the cells were centrifuged for 1 minute at 21,130 rcf to pellet the cells and cell debris, and the supernatant containing the progeny phage lysate was collected and filtered through a 0.45 µm filter to remove any residual bacterial cells. Progeny and parent phages were stored at 4°C.

Cryo-FIB-ET

Cells expressing gp210-GFPmut1 were grown for 3 hours in a humidor at 30°C on 10 agarose pads (1% arabinose, 1% agarose, 25% LB). 10 µl of high titer ΦKZ lysate was added to

the pads and incubated for another hour before 25 µl of 25% LB was added at RT and the cells were gently scraped from the pads using the bottom of an eppendorf tube and collected for plunging.

A custom-built manual vitrification device (Max Planck Institute for Biochemistry, Munich) was filled with liquid nitrogen (LN_2) to maintain a and an ethane/propane gas mixture was condensed to a liquid in a LN_2-cooled cup in the device. Room humidity was kept around 30% to mitigate water contamination. Holey carbon-coated QUANTIFOIL® R2/1 copper grids were glow discharged (0.2 mbar, 20 mA, 60 sec) 30 minutes prior to plunging using a Pelco easiGlow™ system. 7 µL of concentrated infected cells were put on the carbon side of each grid. Samples were blotted with Whatman filter paper No. 1 to remove excess liquid and plunge-frozen in the liquid ethane/propane to be vitrified.

Grids were mounted into cryo-FIB AutoGrids (TFS) and milled as previously described using an Aquilos (TFS) dual-beam scanning electron microscope under cryogenic conditions (*50*). Briefly, areas of interest were rough-milled with an ion-beam current of 0.1-0.50 nA, followed by fine milling and polishing at 30-50 pA. One grid was prepared for each condition to yield two lamellae for the wild-type infection and four lamellae for infected host cells expressing the gp210-GFPmut1 fusion.

Lamellae were imaged under cryogenic conditions using a Titan Krios (TFS) transmission electron microscope operated at 300 kV and equipped with a K2 direct electron detector (Gatan) mounted post a Quantum 968 imaging filter (Gatan). The microscope was operated in EFTEM using a 20 eV slit-width and the detector operated in counting mode. Data collection was performed semi-automatically using SerialEM-v3.8.0 (*51*). Tilt-series were collected following a dose-symmetric scheme starting at the specimen pre-tilt and targeting a tilt-range of +/- 60° in increments of either 2°, 2.5°, or 3°, using a pixel size of either 3.457 Å or 4.265 Å. Exposure times for each tilt-movie were adjusted to maintain constant counts on the detector throughout the tilt-series. The cumulative dose for each tilt-series was usually between ~150-200 e/$Å^2$ and defocus between 5 to 7 µm. For tilt-series of the gp210-GFPmut1 expressing host cells, 14 were acquired at 3.457 Å and 3 at 4.265 Å. For the tilt-series of wild-type host cells, 2 tilt-series were acquired at both pixel sizes.

All tilt-movies were motion-corrected and defoci estimated using Warp-v1.09 (*52*). Tilt-series alignment was performed by patch-tracking using Etomo from IMOD-v4.10.28 (*53*). Tomograms were reconstructed using Warp-v1.09 with the deconvolution filter applied to those used for visualization.

For each condition, ribosome template-matching was performed using Warp-v1.09 using EMD-25183 filtered to 50 Å as a reference and results curated with Cube (https://github.com/dtegunov/cube). Unless otherwise specified, all conditions were aligned and classified similarly using RELION-v3.1.3 (*54*, *55*). Initially selected subtomograms were reconstructed at 12 Å per pixel and refined against the template-matching reference. Reference-free 3D-classification without alignment was then performed to remove false-positives, followed by an additional round of refinement. For further refinement of particle alignment and tilt-series parameters, half-maps were upsampled to the original pixel size and imported into M-v1.0.9 (*56*). Lastly, subtomograms were reconstructed at the original pixel size and subjected to an additional round of reference-free 3D-classification and refinement in RELION-v3.1.3. For the gp210-GFPmut1 expressing host cells, this procedure resulted in a ribosome map at 9.9 Å from 15,439 subtomograms from data collected at 3.457 Å and a map at 13 Å from 4,439 subtomograms from the data collected at 4.265 Å. For wild-type host cells, this procedure

resulted in a ribosome map at 39 Å from 921 subtomograms from the data collected at 3.457 Å and a 13.7 Å map from 6,475 subtomograms from data collected at 4.265 Å.

For the tubular assemblies of the ΦKZ major capsid protein, an initial reference was generated using the MATLAB-v2019b (Mathworks) implementation of Dynamo-v1.1514 (*57*). The surface of an individual, single-layer tube was twice over-sampled assuming a repeat distance of 145 Å (Fig. S4, A and B). Subtomograms were extracted at 12.5 Å per pixel and oriented normal to the surface with random in-plane rotation. These subtomograms were aligned against a smoothed version of the average generated from the unaligned subtomograms (Fig. S4D). The alignment procedure restricted out-of-plane searches to a 60° cone, allowed unrestricted in-plane searches, restricted shifts to 75 Å from the initial position, applied an ad hoc lowpass filter of 40 Å, and C1 symmetry each iteration for 20 iterations. After 15 iterations, the reference ceased to change in subsequent iterations and showed clear hexamer densities with apparent C2 symmetry (Fig. S4, D and E). The reference was shifted to center on a hexamer, cropped, and C2 symmetrized to use for alignment of the remaining subtomograms.

To more precisely define the subtomogram position from the variable tube diameter and layers, the tube axes were first manually traced and refined. For each tubular structure, the start/end points were manually annotated and subtomograms were defined as overlapping segments along the tube axis. The subtomograms were then aligned to a smoothed reference generated from the unaligned subtomograms. The alignment procedure restricted out-of-plane and in-plane rotations to 15°, restricted shifts to 75 Å from the initial position along the tube axis but only 25 Å perpendicular to the axis, applied an ad hoc lowpass filter of 40 Å, and C1 symmetry each iteration for 5 iterations. A moving average using a sliding window of 3 points along the aligned subtomogram positions was computed as the refined tube axis. Radii were determined from line-profiles of Z-projected, rotationally averaged final averages. The tube axis and radii determined through this procedure were used to extract subtomograms as described for the initial reference. Any subtomogram positions outside of lamellae boundaries were discarded prior to alignment.

All subtomograms were aligned against the initial, symmetrized reference generated above while applying a cylindrical mask to exclude adjacent layers in the case of multi-layer tubes (Fig. S4F). This alignment was performed for a single iteration while restricting out-of-plane searches to a 60° cone, restricting in-plane searches to 180°, restricting shifts to 75 Å from the initial position, applying an ad hoc lowpass filter of 40 Å, and C2 symmetry.

Post-alignment subtomograms were subject to a multi-step curation procedure. First, aligned subtomograms which converged to the same position were dereplicated by discarding the one with the lower cross-correlation score. Next, subtomograms with fewer than 5 neighboring subtomograms within 125 to 162.5 Å were discarded. Then, the subtomogram cross-correlation scores were normalized on a per-tomogram basis based on the subtomograms' orientation with respect to the missing-wedge to account for systematically lower scores of these orientations (Fig. S4C), as previously described (*58*). The subtomogram positions were evaluated using the PlaceObject-v2.1.0 plugin (*59*) for UCSF-Chimera-v1.14 (*60*) to determine suitable cross-correlation threshold to exclude misaligned subtomograms. Threshold values typically fell between 0.5-0.6 based on the normalized values. This resulted in 21,121 curated subtomograms.

Subtomogram metadata was converted for further processing with Warp/RELION using dynamo2m-v0.2.2 (*61*). Subtomograms were extracted at 3.457 Å using Warp-v1.0.9 and further refined with C2 symmetry in RELION-v3.1.3 using half-sets composed on a per-tube basis. The final subtomogram average obtained has estimated resolution of 13 Å (Fig. S4K). Final aligned

subtomogram positions were assessed by a neighbor plot (62), which reflected the expected geometry based on the obtained subtomogram average (Fig S4J).

A coordinate model for ΦKZ major capsid protein was predicted using the ColabFold (63) implementation of AlphaFold2 (64). Multiple copies of the resulting model were docked into the final subtomogram average and flexibly fitted into the map using the Namdinator webserver (65). Geometry minimization with restraints generated from the original AlphaFold2 prediction and C2 symmetry constraints was performed using Phenix-v1.19 (66) (Fig. S4G). Rendering of the maps, models, and surface properties was performed using ChimeraX-v1.4 (60).

Isolation and sequencing of ΦKZ mutants resistant to gp210

To obtain ΦKZ mutants that were able to plaque successfully when the host was expressing gp210-GFPmut1, whole plate infections were generated from 100 µl of cells (OD_{600} ~0.4) induced for over an hour with 0.1% arabinose and combined with 10 µl of ΦKZ lysate (10^{12}). Phage adsorption was allowed to proceed while stationary for 10 minutes at room temperature (RT) prior to the addition of 5 ml warm 0.35% LB top agar. The mixture was poured onto an LB plate with 15 µg/ml gentamycin and incubated overnight at 30°C in a humidor. Isolated plaques from each plate were streaked onto a new plate and overlaid with top agar containing 0.1% arabinose and cells expressing gp210-GFPmut1 then incubated overnight. Plaque isolation was repeated twice more and the final isolate was streaked to achieve web lysis. Those plates were soaked with 5 ml chilled phage buffer for 5 hours then collected and centrifuged at 3,220 rcf for 10 minutes at 4°C. The supernatant was filtered through a 0.45 µm Corning membrane filter by syringe, 5 drops of chloroform were added, it was shaken by hand for 2 minutes, then centrifuged again to separate the aqueous phase. Lysate was drawn off the top for standard titers on cells expressing GFPmut1 or gp210-GFPmut1. For mutants displaying resistance to gp210, 2 whole plate infections for each isolate were generated and the lysates collected as described above.

Phage genomic DNA was isolated from each mutant using 10 ml lysate incubated with 5 µl of each RNaseA (100 mg/mL) and DNaseI (20mg/mL) at 37°C for 30 minutes. Then 4 ml of phage precipitant solution (30% PEG 8000, 19.3% NaCl in ddH_2O) was added and incubated overnight at 4°C. Samples were centrifuged at 10,000 rcf 4°C for 20 minutes and the pellets were resuspended in 0.5 ml sterile water for incubation at 5 minutes at RT. Then 2.5 ml of Qiagen Buffer PB was added and incubated at RT for 10 minutes with occasional swirling. The resuspensions were filtered through Qiagen PCR Purification columns and washed twice with Qiagen Buffer PE. The columns were dried with an extra 2 minute spin before 100 µl of 37°C Tris-EDTA buffer was added and allowed to soak for 5 minutes before elution.
Samples were sequenced by the Microbial Genome Sequencing Center (MiGS) in Pittsburgh. Whole genome sequencing was performed using the Illumina NextSeq 2000 platform at a depth of 200Mbp. MiGS provided paired end reads (2x151bp) and reported variations from the Genbank entry for ΦKZ (NC_004629.1).

Image quantitation

To measure differences in DNA content of the phage nuclei using DAPI concentration, cells expressing either GFPmut1 or gp210-GFPmut1 were grown with 1% arabinose and 1 µg/ml DAPI for 3 hours then infected by ΦKZ for 45 minutes. Raw images were analyzed in Fiji 1.53c. The raw integrated density of each phage nucleus was measured by an inscribed circle on the Z slice closest to the middle of the nucleus. Background raw intensity was measured from empty

space next to each infected cell that was measured. Intensity was normalized to the area of the region measured and the background subtracted. A violin plot of these values was generated using Prism 9.2.0 by GraphPad.

Statistical analysis

For EOP statistics, a two-tailed ratio paired t-test was used since the differences between control and treated are larger when the control values are larger. For capsid counts and competition titers, a two-tailed unpaired t-test was used since the groups are unmatched and it is reasonable to assume a Gaussian distribution.

Materials availability

Materials are made available upon request.

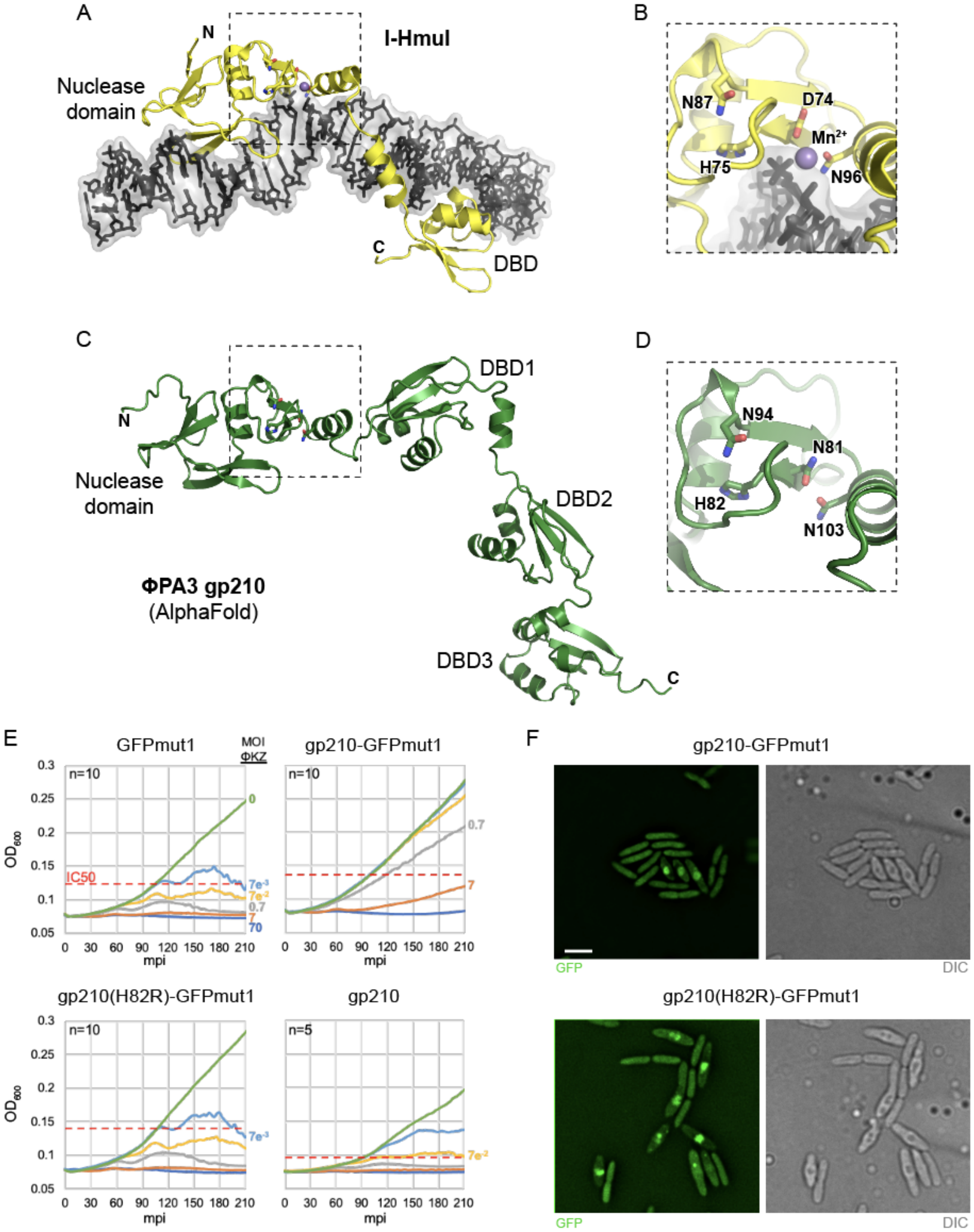

Fig. S1. ΦPA3 gp210 is a putative homing endonuclease.

(A) Structure of the I-HmuI homing endonuclease from phage SPO1 (yellow) bound to its target DNA site (black) (*67*). The protein has two domains: an N-terminal HNH/N nuclease domain, and a C-terminal DNA binding domain (DBD).

(B) Closeup of the I-HmuI active site, with catalytic residues shown as sticks and a bound Mn^{2+} ion shown as a purple sphere.

(C) AlphaFold-predicted structure of ΦPA3 gp210 (green), with predicted N-terminal HNH/N nuclease domain and three C-terminal DNA binding domains (DBD1-3) labeled.
(D) View of the ΦPA3 gp210 active site in the same orientation as panel (B), with putative active site residues shown as sticks.
(E) ΦKZ growth curves measuring OD_{600} of bacteria in liquid culture showed an MOI of $7e^{-3}$ was required to achieve 50% inhibition of cell growth (IC50: red dotted lines) when cells expressed GFPmut1 (N=10). gp210-GFPmut1 increased the IC50 MOI to 7, a 1,000-fold decrease in ΦKZ fitness (N=10). gp210(H82R)-GFPmut1 rescued the IC50 back to $7e^{-3}$ (N=10), while untagged gp210 in the cytoplasm caused only a 10-fold increase in required MOI (N=5).
(F) Field images of ΦKZ infecting *P. aeruginosa* cells expressing either gp210-GFPmut1 or gp210(H82R)-GFPmut1 showing that both GFP fusions (green) are imported into the ΦKZ nucleus. Scale bar is 3 μm.

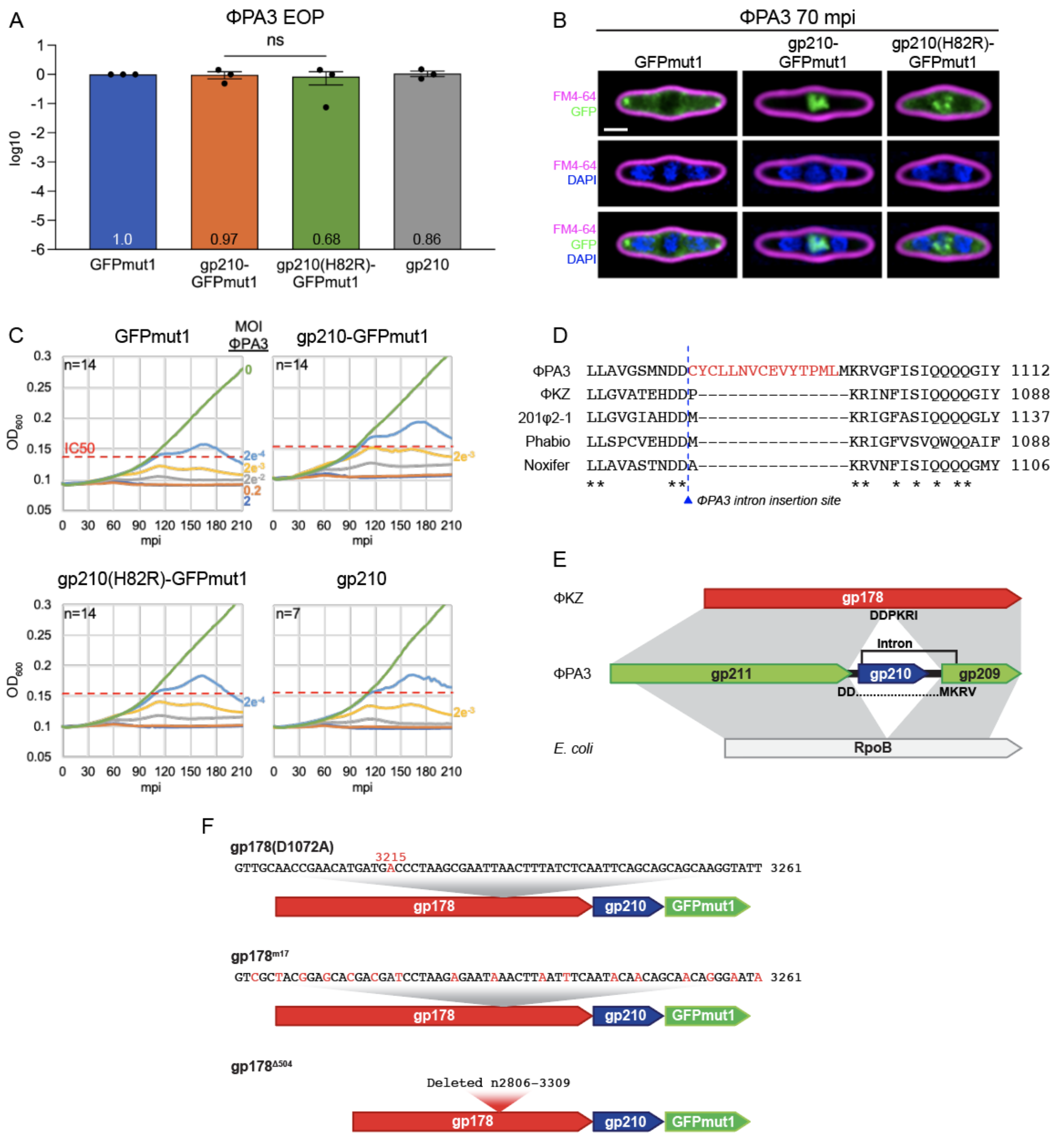

Fig. S2. ΦPA3 is unaffected by gp210. The mobile intron aligns with the uninterrupted ΦKZ gp178 gene.

(A) ΦPA3 is not significantly affected by expression of gp210-GFPmut1 ($p=0.74$), gp210(H82R)-GFPmut1 ($p=0.51$), or untagged gp210 ($p=0.93$). The difference between gp210-GFPmut1 and gp210(H82R)-GFPmut1 is also insignificant ($p=0.42$). Ratio paired t-tests were used to determine p values.

(B) Live fluorescent microscopy of stained ΦPA3 infections in the presence of either GFPmut1, gp210-GFPmut1, or gp210(H82R)-GFPmut1, with no obvious morphological differences between them. Scale bar is 1 μm.

(C) ΦPA3 growth curves demonstrating only a 10-fold decrease in ΦPA3 fitness with the expression of gp210-GFPmut1 or untagged gp210 but no change with gp210(H82R)-GFPmut1.
(D) Protein alignment of *Pseudomonas* jumbo phage RNAPs reveals an extra 15 residues are included in the Genbank annotation of ΦPA3 gp211. Correct splicing is presented in Figure 1E.
(E) Diagram of RNAP subunit genes of nucleus-forming *Pseudomonas* jumbo phages ΦKZ and ΦPA3 aligned with RpoB of *E. coli*.
(F) Nucleotide sequences of the gp178 variants: gp178(D1072A) with nucleotide mutation a3215c, gp178^{m17} with 17 silent mutations intended to disrupt gp210 targeting while maintaining the amino acid sequence, and gp178$^{\Delta 504}$ containing an in-frame deletion of 504 bp as a control for the effects of an upstream ORF in the co-expression with gp210-GFPmut1, which does not contain the region targeted by gp210.

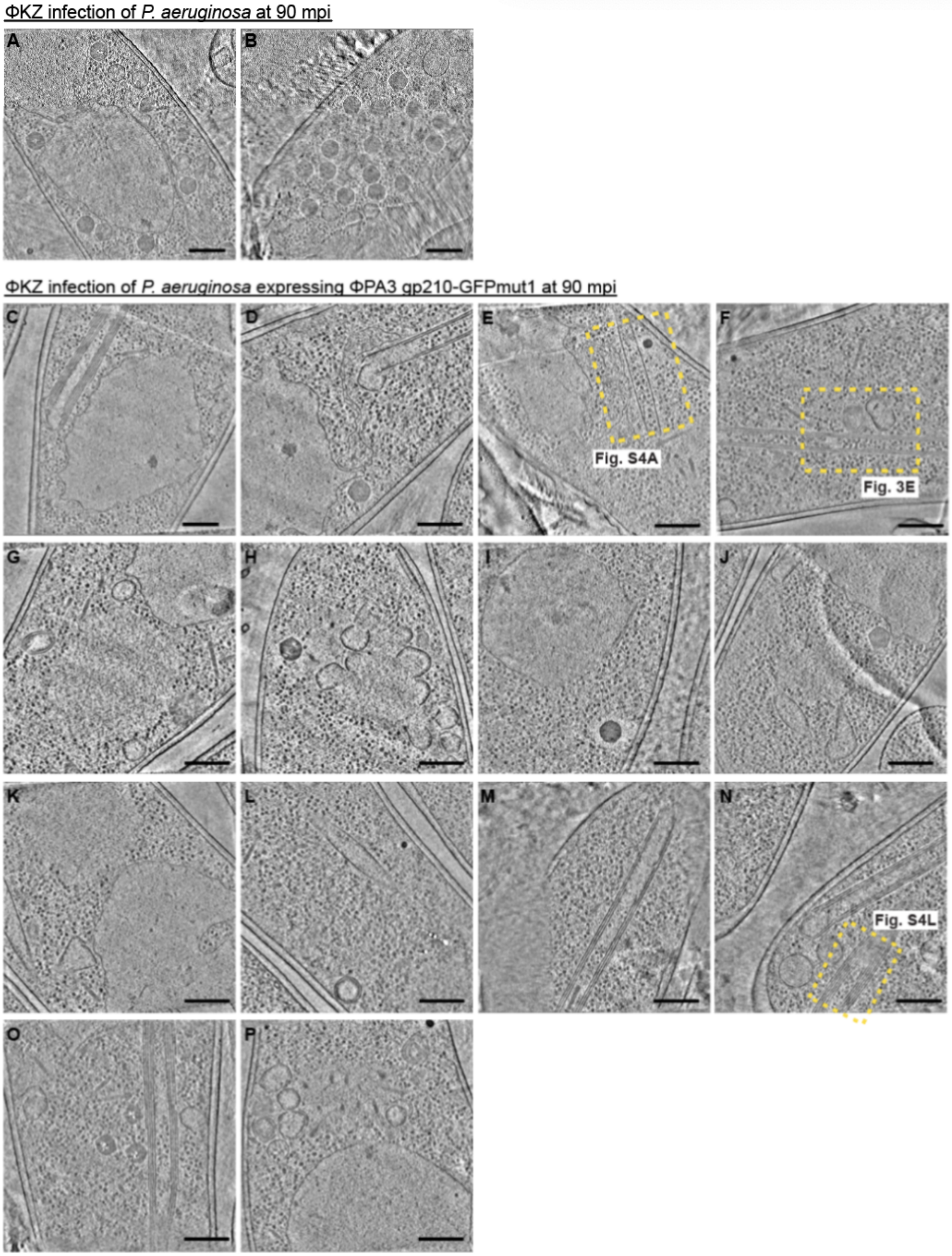

Fig. S3. All collected tomograms of ΦKZ infections.
(A,B) ΦKZ-infected *P. aeruginosa* cells.

(C-P) ΦKZ-infected *P. aeruginosa* cells expressing ΦPA3 gp210-GFPmut1. Regions boxed by yellow dashed lines are enlarged and cropped for display in corresponding Figures S4A, 3E, and S4L. Scale bars: 250 nm.

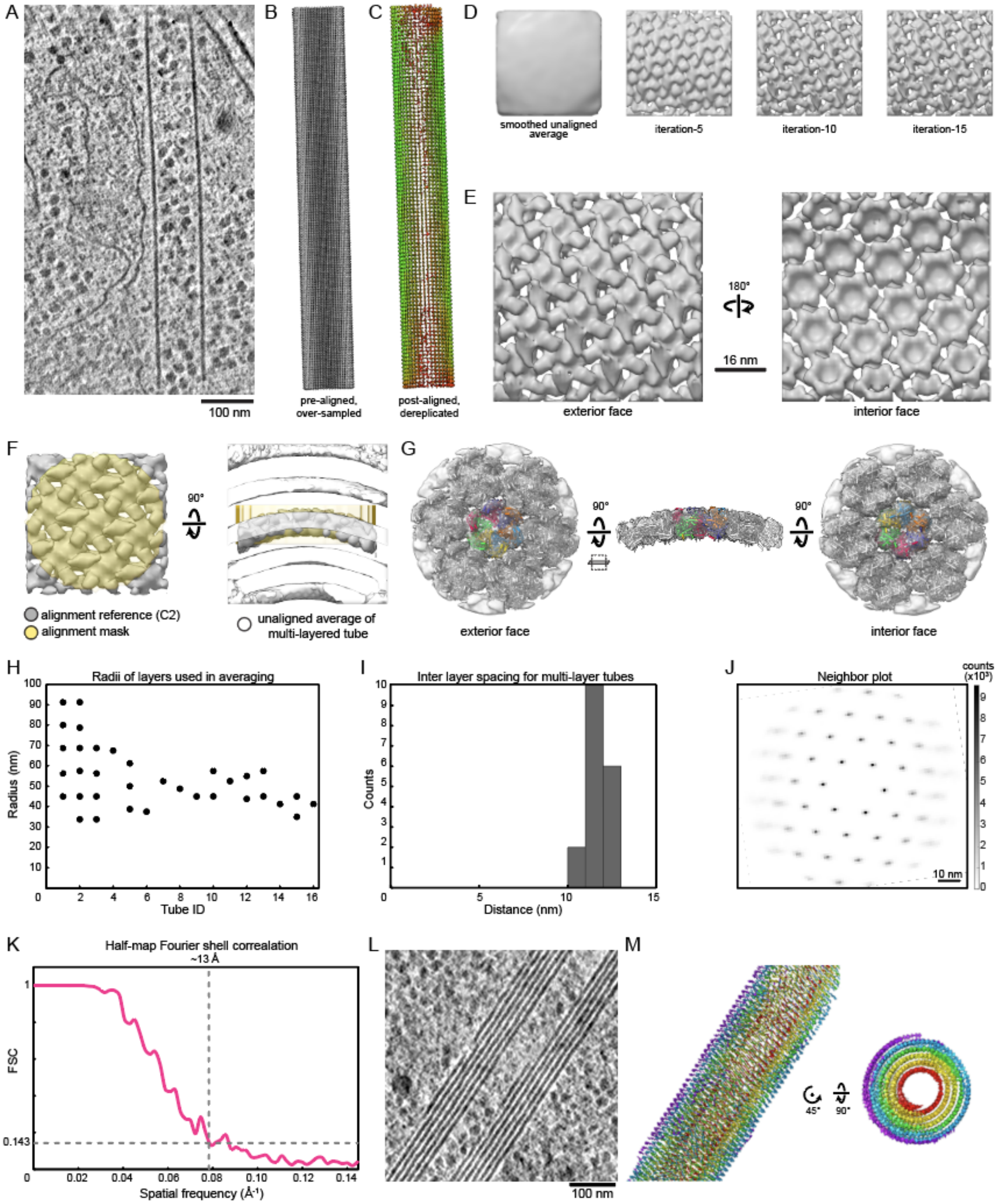

Fig. S4. Subtomogram analysis of tubular assemblies of ΦKZ Major Capsid Protein.
(A) Exemplar single-layer Major Capsid Protein (MCP) tube used to generate an initial average.
(B) Z-arrow lattice plot of over-sampled tube in A pre-alignment.
(C) Same as B post-alignment, dereplicated, and colored red to green for low to high cross-correlation score to the reconstruction, respectively.
(D) *Ab initio* reference at indicated iterations of initial alignment.
(E) Enlarged views of the exterior and interior faces of the *ab initio* reference.

(F) Cropped, shifted reference used to align the remaining data set in grey with alignment mask shown in yellow. Unaligned reconstruction of subtomograms from a multilayer tube is shown white.
(G) Views of the final reconstruction with fitted coordinate model of the MCP. The protomers of the central hexamer are colored individually and surrounding hexamers are colored white.
(H) Plot of observed tube radii.
(I) Histogram of interlayer distances for multilayer tubes.
(J) Neighbor plot of center-to-center hexamer distances of the final aligned subtomogram positions.
(K) Half-map Fourier shell correlation for the final reconstruction.
(L) Tomogram of an aberrant ΦKZ Major Capsid Protein (MCP) assembly observed during expression of ΦPA3 gp210-GFPmut1 in the host cell.
(M) Views of the lattice plot depicted as Y-arrows for aligned positions extracted from the region shown in L. Colors indicate the initially assigned tube layers of the sub tomograms from inner (red) to outer (violet).

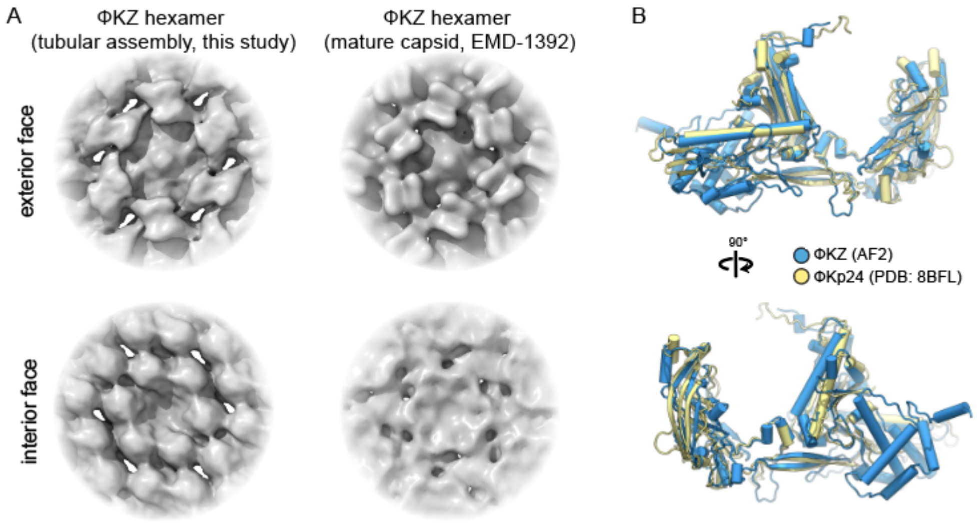

Fig. S5. Comparison of immature ΦKZ and published mature jumbo phage major capsid protein (MCP) structures.
(A) Left, exterior and interior faces of the immature ΦKZ MCP density map obtained in this study by subtomogram analysis of the tubular assemblies. Right, exterior and interior faces of the mature ΦKZ density map (EMD-1392) obtained from single-particle analysis of purified virions *(68)*.
(B) Structural alignment of the AlphaFold2 predicted model of the immature ΦKZ MCP (blue) with the experimentally determined structure of the ΦKp24 MCP (yellow, PDB: 8BFL) *(69)*.

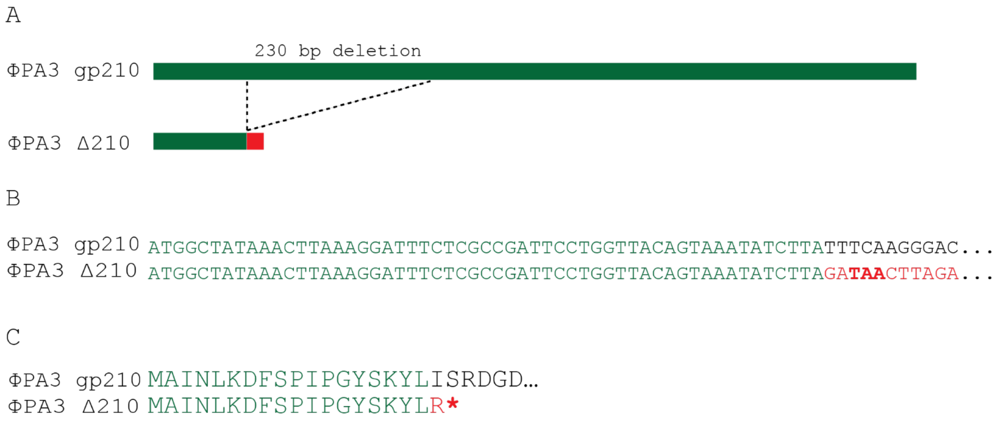

Fig. S6. Gp210-deficient ΦPA3 (ΦPA3Δ210) isolated with Cas13a guide targeting gp210 catalytic region.
(A) Cas13a escape mutant ΦPA3Δ210 exhibits a 230 base pair deletion leading to a frame shift and early stop codon after amino acid 19.

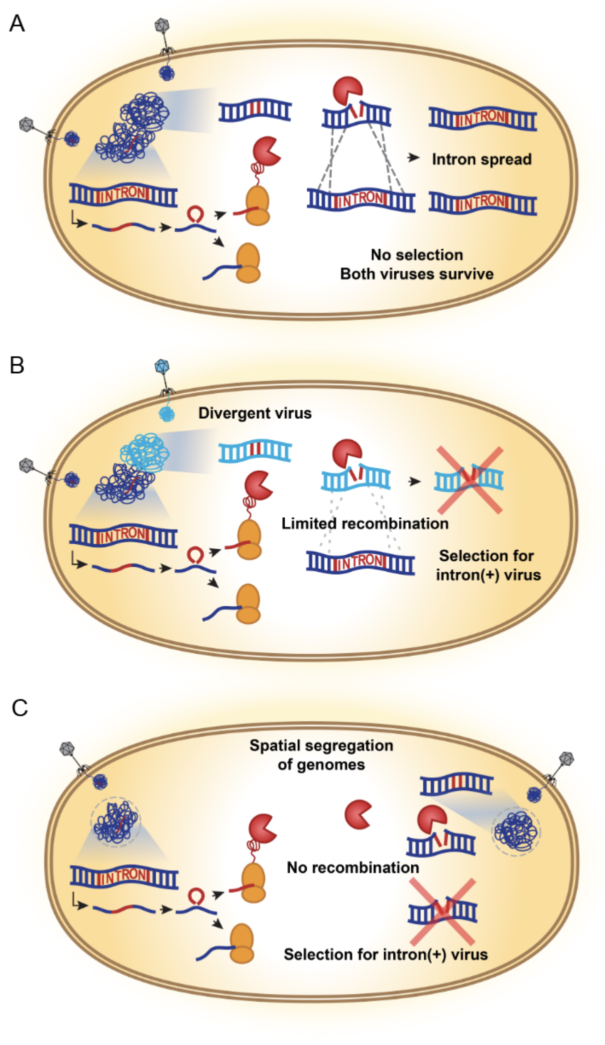

Fig. S7. Models of homing endonuclease-mediated interference competition for phages outside of the *Chimalliviridae* family.

(A) Theoretical model of mobile intron spread during co-infection between any related viruses that replicate their genomes freely in the cytoplasm of the host.

(B) Theoretical model of mobile intron competition between divergent viruses that can physically mix their genomes but sequence divergence has reduced the efficiency of homologous recombination while a highly conserved site is still targeted by the homing endonuclease. This results in a selective advantage for the intron(+) virus.

(C) Theoretical model of mobile intron competition between closely related viruses that display subcellular genetic isolation. Whether spatially separated in distinct replication factories or physically isolated by a barrier such as the phage nucleus, the homing endonuclease can target the essential gene in the intron(-) virus but the repair template containing the intron that interrupts the target site is sequestered. This results in the destruction of the intron(-) virus and a strong selective advantage for the intron(+) virus.

Mathematical modeling of phage competition.

Here we explore how gp210 is expected to affect competition between ΦPA3 and ΦKZ. Using population growth equations, we show that whether ΦPA3 outcompetes ΦKZ depends on whether ΦPA3 possesses gp210, the multiplicity of infection, and the ratio of ΦPA3 to ΦKZ.

To start, we will extrapolate what is expected to happen if we were able to continue the gp210 cost experiment (ΦPA3 ΦKZ co-infection and quantification), the one in which we measured the 3.7-fold reduction in ΦKZ growth due to gp210 (Fig. 4, A and B), for multiple generations. In the experiment, ΦKZ was rare relative to ΦPA3, ~10^7 and ~10^9 plaque forming units, respectively. Given the ΦPA3 multiplicity of infection (MOI) of 10:1, virtually all cells were infected by ΦPA3 and only a fraction by ΦKZ. To model the population dynamics resulting from their competition, we will start by modeling their exponential growth.

Population growth of ΦPA3 with or without gp210 can be modeled using the following equation, assuming there is no cost to harboring the homing endonuclease:

$$N_{P,t} = N_{P,0}e^{rt} \qquad \text{equation 1}$$

Where N_P indicates population size of ΦPA3, r is its exponential growth rate of ΦPA3, and t is time in generations (a single infection). A single ΦPA3 infection yields 100 ($N_{P,0}$=1, $N_{P,1}$=100, t=1), so r=ln(100/1)/1 or ~4.6.

Growth of ΦKZ against ΦPA3 without gp210 can be described as follows:

$$N_{K,t} = N_{K,0}e^{rct} \qquad \text{equation 2}$$

'*c*' included in equation 2 is a coefficient set by how much faster or slower ΦKZ grows relative to ΦPA3. Our best estimate of this is ~1.056. This value was derived from a previous paper (*15*) where ΦKZ infections produced 1.3 times as much DNA as ΦPA3 per infection. Since the burst size of a single infection of ΦPA3 is 100, ΦKZ will be 130. We can solve for c for a single infection using $N_{K,t}$ = 130, $N_{K,0}$ = 1, t=1, r=4.6. This equation shows that as long as c>1, then ΦKZ will grow faster than ΦPA3, and the rare ΦKZ will be able to invade and take over. Under these conditions, c=1.056, so ΦKZ will outcompete ΦPA3 (Fig. S8).

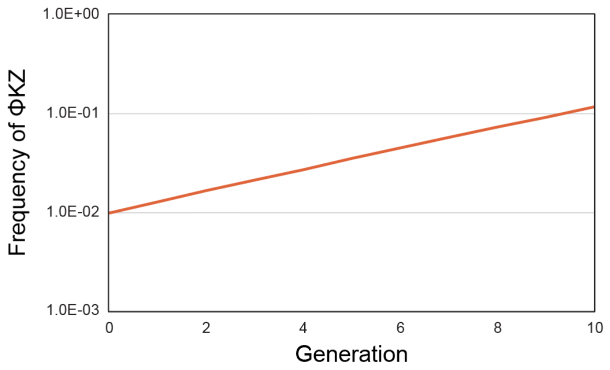

Fig. S8. ΦKZ increases in frequency relative to ΦPA3 over time because of ΦKZ's higher growth rate.

Growth of ΦKZ during co-infection with ΦPA3 containing gp210 can be calculated as follows:

$$N_{K,t} = N_{K,0}e^{(\frac{rc}{h})t} \qquad \text{equation 3}$$

'h' was included in equation 3 to account for the growth rate penalty caused by co-infecting with ΦPA3 that has gp210. We modeled the effect of gp210 as a growth penalty rather than an independent death rate because when measuring gp210's effect, we found that it did not stop ΦKZ from growing, but slowed it down by 3.7 fold. The variable h has to equal values above 1, since values below 1 would provide a fitness benefit by gp210 to ΦKZ, which seems unlikely. A value of one is the scenario where gp210 is absent from the genome (Fig. S8). We can solve for h, given the scenario where a single ΦKZ was reduced by 3.7 times yielding approximately 35 progeny instead of 130 progeny; $N_{K,t}$=35, $N_{K,0}$=1, t=1, r=4.6, and c=1.056. In this scenario, h=1.37 (Figure S9).

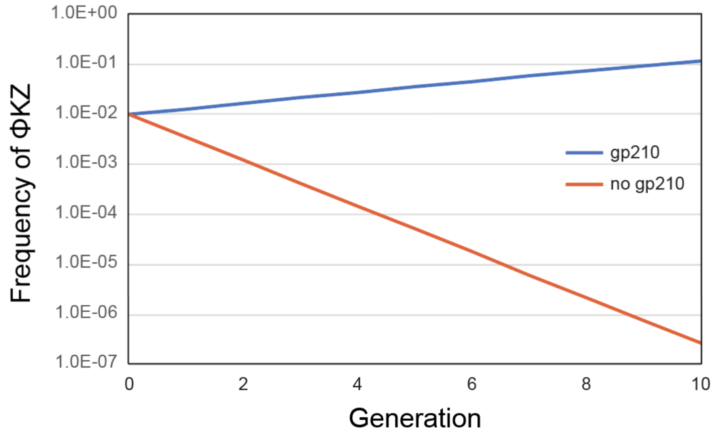

Fig. S9. ΦKZ is less competitive when ΦPA3 possesses gp210.

Equation 3 is for a highly unlikely scenario where every single cell infected by ΦKZ is also infected by ΦPA3. To model more likely scenarios, where there's a mixture of single and cross-species co-infections, we can write an equation that predicts population growth based on these two types of infections, where the fraction of cross-species co-infections is m.

$$N_{K,t+1} = mN_{K,t}e^{(\frac{rc}{h})t} + (1-m)N_{K,t}e^{rct} \qquad \text{equation 4}$$

Using this discrete time model, we can predict the dynamics of ΦKZ under a range of scenarios (Fig. S10). The graph in Figure S10 shows a scenario when ΦKZ and ΦPA3 are initially present in equal numbers, and the frequency of co-infection is either 5%, 25%, or 50%.

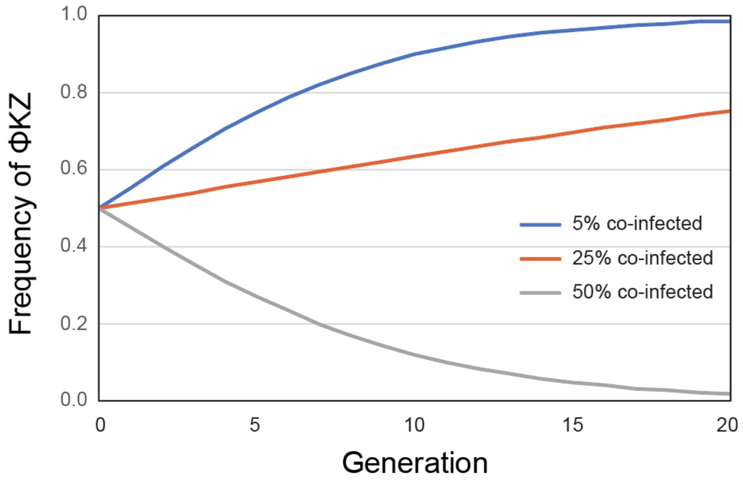

Fig. S10. Whether ΦKZ is more competitive than ΦPA3 depends on the frequency of cross-species co-infections.

This exercise shows that the outcome of competition between ΦKZ and ΦPA3 depends on the probability of cross-species co-infection (m), which will be a function of MOI and the ratio of ΦKZ and ΦPA3. The co-infection rate at which the relative fitness of ΦKZ and ΦPA3 is predicted to be equal is approximately 0.31. In highly competitive conditions, co-infection at 31% or greater is likely to occur in nature and therefore gp210 would be expected to play a role in phage competition and maintaining the competitively inferior phage.

FM 4-64	Thermo Fisher Scientific	Cat#T13320
DAPI (4',6-Diamidino-2-Phenylindole, Dihydrochloride)	Thermo Fisher Scientific	Cat#D1306
Gentamycin sulfate	Sigma-Aldrich	Cat#G1914
T4 DNA ligase	New England Biolabs	Cat#M0202L
Phusion High-Fidelity DNA Polymerase	New England Biolabs	Cat#M0530L
Deoxynucleotide (dNTP) Solution Mix	New England Biolabs	Cat#E5520S

Table S1. Chemicals
